# Thyroidectomy-Related Dysphagia: A Systematic Literature Review

**DOI:** 10.3390/medicina62030440

**Published:** 2026-02-26

**Authors:** Eleni Litsou, Chrissa Sioka, Konstantinos Mpakogiannis, Labrini Magou, Polyxeni Fakitsa, Alexandros Giannakis, Sakkou Sissy Foteini, Fotios Fousekis

**Affiliations:** 1 Department of Otorhinolaryngology, Head and Neck Surgery, University Hospital of Ioannina, 45500 Ioannina, Greece; 2 Department of Nuclear Medicine, University Hospital of Ioannina, 45500 Ioannina, Greece; csioka@yahoo.com; 3 Department of Gastroenterology, Alexandra Hospital, 11528 Athens, Greece; kostismpakogiannis@gmail.com; 4 Department of Physical Medicine and Rehabilitation, University Hospital of Ioannina, 45500 Ioannina, Greece; labmagou@gmail.com (L.M.); j.fakitsa@gmail.com (P.F.); 5 Department of Neurology, University Hospital of Ioannina, 45500 Ioannina, Greece; papadates@gmail.com; 6 Department of Internal Medicine, University Hospital of Ioannina, 45500 Ioannina, Greece; sissy_sakkou@hotmail.com; 7 Department of Gastroenterology and Hepatology, University Hospital of Ioannina, 45500 Ioannina, Greece; fotisfous@gmail.com

**Keywords:** thyroid surgery, thyroidectomy, dysphagia, deglutition disorders, swallowing disorders, quality of life

## Abstract

*Background and Objectives*: Dysphagia is a frequently reported symptom among patients undergoing thyroidectomy, yet its incidence, underlying mechanisms, and temporal progression remain insufficiently clarified. The aim of the present systematic review was to synthesize the existing literature on the occurrence and evolution of swallowing disorders following thyroidectomy, without restriction regarding the extent of surgery, surgical approach, indication, or concomitant complications. *Materials and Methods*: A systematic literature review, according to PRISMA guidelines, was conducted in the electronic databases PubMed, MEDLINE, and SciELO, using the terms “dysphagia”, “deglutition disorder”, “swallowing disorder”, “thyroid surgery” and “thyroidectomy” in the appropriate combinations. A narrative synthesis of the results followed. *Results*: 31 eligible studies encompassing a total of 64,123 patients were included in the systematic review and analyzed concerning their type, sample, follow-up and results regarding thyroidectomy-related dysphagia. Data regarding pre- and postoperative dysphagia were extracted and compared. Both subjective patient-reported outcomes and objective assessments were considered. Reported preoperative dysphagia incidence varied widely (3.3–77.8%), with a pooled mean of approximately 25%. Dysphagia rates increased significantly within the first 1–2 postoperative weeks but generally declined to near preoperative levels by 2–3 months, with further improvement observed up to 4–6 months. Several factors were associated with persistent or more severe dysphagia, including the extent of surgery, older age, surgical techniques, central or lateral lymph node dissection, and the need for adjuvant therapies such as radioactive iodine or external beam radiotherapy. *Conclusions*: Dysphagia after thyroidectomy appears as a common but typically transient symptom, with the highest incidence occurring in the immediate postoperative period and a progressive return to baseline within three months. Although most patients experience improvement, a subset may report persistent symptoms with measurable impact on quality of life. Methodological heterogeneity, variability in symptom assessment tools, and limited long-term follow-up restrict the strength of available evidence. Standardization of outcome measures and longer follow-up periods are needed to achieve more reliable and generalizable conclusions.

## 1. Introduction to the Systematic Review

Thyroidectomy is currently a very common surgical procedure and, more specifically, the most frequently performed procedure involving the endocrine glands. Swallowing disorders are now a recognized complication of this particular surgery, reported both after intraoperative nerve injury and after uncomplicated operations. However, it is worth noting that symptoms suggestive of dysphagia have also been reported after other surgeries, a fact that tends to dissociate thyroidectomy from being the sole operation potentially causing the onset of such symptoms. Furthermore, since in quite a few cases the patients-reported symptoms are not confirmed by objective examinations, several authors refer to these cases as “post-thyroidectomy syndrome.”

The symptoms associated with dysphagia after thyroidectomy, as well as their severity, vary considerably from patient to patient. They may include pain or discomfort when swallowing, a choking sensation, a feeling of a foreign body, the sensation of a “lump” in the throat, coughing, and others. Explaining the causes of these symptoms is not always possible, even after a series of examinations to investigate dysphagia.

Finally, dysphagia often appears preoperatively in patients with thyroid diseases and constitutes one of the possible indications for surgery in these cases. The scientific surgical community’s interest in the subject lies in the fact that such symptoms seem to negatively affect patients’ quality of life, even when the procedure is not performed in the context of malignancy. The impact of dysphagia on both daily life and the broader social life of patients is the reason they often seek medical assistance.

In addition to patient-reported symptoms, several studies have attempted to objectively assess swallowing function following thyroidectomy using instrumental methods, including videofluoroscopic swallowing studies, fiberoptic endoscopic evaluation of swallowing, high-resolution esophageal manometry, electromyography, and ultrasound-based kinematic analysis. These objective approaches have provided insight into postoperative alterations in laryngeal elevation, hyoid bone displacement, pharyngoesophageal segment pressure, and esophageal motility, particularly during the early postoperative period. However, the use of such tools remains inconsistent across studies, and objective findings do not always correlate with subjective symptom severity [1,2,3,4,5,6].

## 2. Materials and Methods

### 2.1. Search Strategy

The present systematic literature review on the occurrence of swallowing disorders in thyroidectomy, following the recently revised PRISMA guidelines for systematic reviews (File S1), was conducted in the electronic databases PubMed, MEDLINE, and SciELO, using the combination of keywords: [“dysphagia” OR “swallowing disorder” OR “deglutition disorder”] AND [“thyroidectomy” OR “thyroid surgery”]. These terms were chosen as umbrella terms, which consequently facilitated the inclusion of the broadest possible spectrum of relevant published studies. Duplicates were removed from the preliminary compilation of studies. Subsequently, the remaining articles were reviewed twice. Firstly, two independent reviewers (E.L. and M.K.) screened titles, abstracts, and full texts according to the eligibility criteria. The final evaluation of the process was confirmed by a third independent investigator (F.F.). Discrepancies were resolved by consensus and inter-rater agreement. The search was performed between August and September 2025.

### 2.2. Inclusion and Exclusion Criteria

The criteria by which the articles retrieved from the search were deemed eligible for inclusion in the study were as follows:

✓ Research focusing on thyroidectomy performed on human subjects.
✓ Swallowing disorder had to be reported at least at one time point after thyroidectomy and expressed as an absolute number of patients.
✓ Both studies employing patient-reported assessments of dysphagia and those utilizing objective diagnostic methods for swallowing disorders were incorporated.
✓ According to the definition of dysphagia in the literature, based on the symptoms through which it manifests, the present review also included studies that did not explicitly use the terms “dysphagia” or “swallowing disorder,” but described symptoms such as the sensation of a “lump,” a foreign body, or any other related discomfort.
✓ Both prospective and retrospective studies, randomized or non-randomized, were included, regardless of the number of patients enrolled, whether the condition was benign or malignant, the extent of surgery (total thyroidectomy or lobectomy), the surgical technique (open, robotic, or endoscopic procedures), or the language of the text.

Conversely, from the outset, the following were excluded:

❖ Articles that were previous reviews, case reports, animal studies, or those that could not be retrieved in full.
❖ Studies that used questionnaire-based rating scales but did not report the absolute number of patients presenting the disorder, as their results were not comparable with those of other studies.
❖ Articles that did not provide information on this specific postoperative disorder.
❖ Studies involving patients with comorbidities capable of explaining the symptoms independently of thyroid disease (e.g., gastroesophageal reflux, neurological disorders).

Outcome assessment included both subjective patient-reported measures and objective instrumental evaluations of swallowing function. Subjective assessment was performed using validated or author-modified questionnaires, such as the Swallowing Impairment Score (SIS), SWAL-QOL, ThyPRO, and visual analog scales. Objective assessment methods, when available, included videofluoroscopic swallowing studies, fiberoptic endoscopic evaluation of swallowing, ultrasound evaluation of hyoid and laryngeal movement, electromyography, and high-resolution esophageal manometry. Due to methodological heterogeneity, objective data were narratively synthesized rather than quantitatively pooled [1,2,3,4,5,6].

### 2.3. Risk of Bias Assessment (ROBINS-I)

Risk of bias in non-randomized studies was assessed using the ROBINS-I tool (Table 1), while randomized controlled trials (RCTs) were evaluated using principles aligned with the Cochrane RoB 2 tool. Overall, studies showed a moderate to serious risk of bias, primarily due to confounding and outcome measurement.

Important confounders—such as preoperative swallowing status, extent of surgery, nerve monitoring, anesthesia-related factors, and adjuvant therapies—were inconsistently measured or adjusted for, limiting causal inference. Selection bias was common because of single-center designs, unclear recruitment, and lack of appropriate control groups.

Outcomes were mainly assessed using subjective, often non-validated patient-reported questionnaires, with limited use of objective measures, resulting in a high risk of detection bias. Blinding was rarely reported. Several longitudinal studies inadequately addressed missing data and loss to follow-up, increasing attrition bias. Selective reporting was suspected when absolute dysphagia rates or complete time-point data were not provided.

RCTs generally had lower risk of bias but were limited by small sample sizes, short follow-up, and incomplete blinding. Overall, these methodological limitations reduce confidence in estimates of post-thyroidectomy dysphagia and highlight the need for standardized outcomes and better-controlled prospective studies.

## 3. Results

### 3.1. Search Results

**Figure 1:**
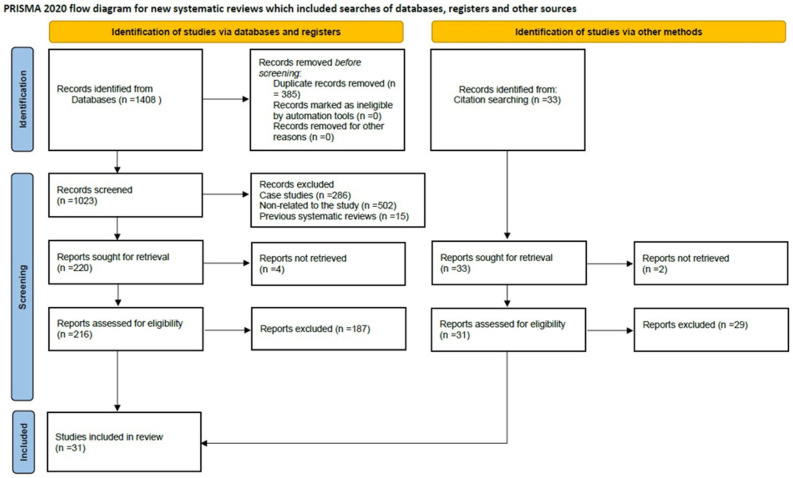
PRISMA flowchart of the studies included in this systematic review [7].

The initial search identified 1408 articles from the PubMed, MEDLINE, and SciELO databases. The removal of duplicate publications (n = 385) followed by those deemed irrelevant to the research content based on their title and abstract (n = 502), case reports (n = 286), and pre-existing reviews (n = 15) first excluded 1188 articles. Of the remaining articles (n = 220), four reports could not be retrieved. Thus, 216 reports were assessed for eligibility. After reading them, 187 articles were excluded as they did not meet the criteria of the present study. Only 29 studies were found to meet the inclusion criteria. Two more were retrieved after thorough scanning of the references of the above. Eventually, 31 studies were included in the systematic review. Search and screening results are shown in the PRISMA flowchart (Figure 1).

From the articles ultimately included, data were extracted regarding the type of study conducted and its time frame, demographic data of the study populations, and preoperative and postoperative data related to the occurrence of dysphagia. A comparison of these data then followed. Of these, 64.5% (n = 20) were prospective in design, 12.9% were retrospective (n = 4), while only 9.7% (n = 3) were randomized controlled trials. In 12.9% (n = 4), the methodology for symptom assessment or data collection was either insufficiently described or entirely unclear. An overview of the basic characteristics of each study is presented in Table 2.

### 3.2. Study Population and Design Characteristics

- A total of 64,123 patients were included.
- The mean patient age was approximately 45 years, with one notable outlier affecting distribution.
- There was a predominance of female patients, consistent with thyroid disease epidemiology.
- There was wide variation in sample sizes (ranging from small case series to large multicenter studies).
- Studies showed broad geographic representation (USA, Brazil, Italy, China, and others).

### 3.3. Preoperative Dysphagia: Prevalence and Characteristics

- Reported incidence varies widely (3.3–77.8%), reflecting substantial heterogeneity.
- Variability is largely attributable to non-standardized assessment methods (questionnaires, interviews).
- Symptoms are often intermittent, mild, and not always functionally limiting.
- Many patients report multiple swallowing-related complaints.
- Dysphagia is a significant factor influencing the decision for surgery, despite inconsistent severity.
- Only 11 of 31 studies reported preoperative dysphagia data, indicating probable under-recognition.

### 3.4. Factors Associated with Preoperative Dysphagia

#### 3.4.1. Laryngeal Function

- Dysphagia was observed in patients both with and without laryngeal mobility abnormalities.
- This suggests mechanisms beyond structural or neurological impairment.

#### 3.4.2. Sex-Related Anatomical Differences

- Men demonstrate greater laryngeal range of motion on ultrasound.
- There were differences attributed to anatomical variation in thyroid cartilage angle (≈90° in men vs. ≈120° in women).
- Clinical relevance remains uncertain.

#### 3.4.3. Thyroid Size and Goiter Extension

- There was no consistent association between thyroid gland size and dysphagia.
- Substernal goiters did not show higher dysphagia rates compared with overall averages.

### 3.5. Surgical Approaches and Indications

- The majority of studies focused on conventional open thyroidectomy.
- Smaller numbers examined endoscopic, robotic, MIVAT, or combined techniques.
- Studies included total, subtotal thyroidectomy and lobectomy for both benign and malignant disease.
- Postoperative dysphagia incidence was not consistently stratified by surgical type or indication.

### 3.6. Postoperative Dysphagia: Temporal Pattern

- Symptoms typically increase early postoperatively.
- They peak within the first postoperative week.
- Symptoms remain elevated during the first month, with gradual improvement after 2 weeks.
- Return to preoperative levels occurs by approximately 3 months.
- Limited long-term data are available; isolated late symptom increases lack baseline comparison.

### 3.7. Outcome Measurement Tools

- Structured questionnaires were frequently used.
- The Swallowing Impairment Score (SIS) was the most commonly applied tool.
- Objective assessments were inconsistently employed.

### 3.8. Role of Surgical Complications

#### 3.8.1. Uncomplicated Thyroidectomy

- Dysphagia was commonly reported despite intact recurrent laryngeal nerves.
- This indicates that dysphagia can occur independently of overt nerve injury.

#### 3.8.2. Possibly Complicated Thyroidectomy

- Includes transient nerve paresis or unspecified nerve status.
- Higher dysphagia incidence was observed at one month postoperatively.
- Early postoperative dysphagia was more frequent in uncomplicated cases.

### 3.9. Comparative Findings and Limitations

- Conflicting results were reported regarding the impact of nerve injury and surgical technique.
- Small number of comparative studies limits interpretability.
- Evidence was insufficient to draw definitive conclusions regarding causation or prevention.

## 4. Discussion

This systematic review evaluated the occurrence, progression, and determinants of swallowing disorders following thyroidectomy. Unlike prior reviews, this analysis encompassed all types of thyroid surgery—total or partial, open, endoscopic, robotic, or minimally invasive—regardless of the underlying diagnosis or concomitant complications, allowing a comprehensive assessment of postoperative dysphagia across diverse clinical settings.

Patients most commonly report dysphagia as a sensation of a “lump” or foreign body, difficulty clearing the larynx, throat dryness, or pain during swallowing [1,31]. Interestingly, subjective complaints often exceed the frequency of objectively detectable abnormalities, suggesting a multifactorial etiology that includes mechanical trauma, postoperative pain, tissue adhesions, psychosomatic factors, or neural injury, particularly to the recurrent or superior laryngeal nerves [13,19,20].

Across the literature, dysphagia generally peaks during the first one to two postoperative weeks and progressively declines to preoperative levels by 2–3 months, with further improvement by 4–6 months [1,2,10,11,13,19,20,27]. Objective assessments using the Swallowing Impairment Score (SIS) or videofluoroscopy confirm early postoperative impairment in laryngeal motility and hyoid excursion, with gradual recovery over the first three months [1,4,6,19,20,22]. Notably, patients with preoperative laryngeal mobility impairment may experience more severe and prolonged symptoms [1,19]. Although most patients recover, some reports describe persistent dysphagia extending into the long-term postoperative period, even years after surgery [9,13,16]. Conversely, patients presenting with preoperative dysphagia often benefit from thyroidectomy, particularly in cases of substernal goiters [3,10,11,12,15,33,34].

Age and sex have been explored as potential risk factors. Older age may correlate with worse early postoperative symptoms in some studies [19,30], but the evidence is inconsistent [4]. Female sex and lower psychological well-being have been associated with increased subjective complaints preoperatively [29], highlighting the interplay of psychosocial factors in symptom perception.

Objective assessment of swallowing function after thyroidectomy has demonstrated measurable, predominantly transient impairments in laryngeal elevation, hyoid excursion, and pharyngoesophageal segment dynamics. Studies employing videofluoroscopy and kinematic analysis have confirmed early postoperative pharyngeal phase abnormalities with gradual recovery within the first three postoperative months. High-resolution esophageal manometry has shown postoperative alterations in upper esophageal sphincter pressure and esophageal motility, particularly in patients with large goiters, with significant improvement over time. Ultrasound-based studies have further identified reduced hyoid bone displacement during swallowing in the early postoperative period. Importantly, objective findings do not consistently parallel patient-reported symptom severity, underscoring the multifactorial and partially subjective nature of post-thyroidectomy dysphagia [1,3,4,5,6].

The extent of surgery, particularly total thyroidectomy, and procedures involving central or lateral lymph node dissection is consistently associated with higher rates of postoperative dysphagia [16,26,28,29]. The comparison between total thyroidectomy and lobectomy yields mixed results; some studies show higher symptom scores with total thyroidectomy in the first three months postoperatively, reflecting transient laryngeal mobility impairment [5,28]. Minimally invasive and robotic techniques offer potential advantages in reducing tissue trauma, adhesions, and scarring, potentially mitigating dysphagia [8,14,18]. However, studies comparing open, endoscopic, and robotic thyroidectomy demonstrate heterogeneous outcomes, likely due to differences in access routes, trocar positioning, and surgeon experience [17,21,23,24,25,32,35]. Current evidence does not definitively confirm superiority of minimally invasive techniques in improving swallowing function, emphasizing the need for large-scale, controlled trials. Technical modifications, including the subfascial versus subplatysmal approach, selective ligation of superior thyroid vessels, and the use of anti-adhesion materials, may influence postoperative outcomes, though the evidence remains limited [19,36,37,38]. Intraoperative nerve monitoring reduces the incidence of postoperative swallowing complaints [16]. From an anesthesiological perspective, strategies that minimize airway trauma—such as using smaller endotracheal tubes, flexible laryngeal masks, intraoperative cuff pressure monitoring, or intravenous lidocaine—have been shown to reduce early postoperative dysphagia [39,40,41,42]. Similarly, perioperative administration of corticosteroids may further mitigate symptoms [43]. Studies related to modifications in treatment practice, perioperative strategies or technical equipment of thyroidectomy in order to reduce postoperative dysphagia are presented in Table 3.

Radioactive iodine and external beam radiotherapy, while essential in oncologic management, may exacerbate postoperative dysphagia [44,45]. These findings underscore the importance of balancing therapeutic efficacy with functional outcomes when planning adjuvant treatment.

Dysphagia has a substantial negative effect on health-related quality of life, influencing both physical and psychological domains [3,46,47]. Even when symptoms are mild or transient, they may reduce patient satisfaction and motivation for surgery, highlighting the need for comprehensive preoperative counseling and long-term monitoring.

Overall, postoperative swallowing dysfunction after thyroidectomy is multifactorial, influenced by patient characteristics, surgical extent, technique, perioperative management, and adjuvant therapies. Recognition of the temporal pattern of recovery, coupled with individualized surgical planning, technical modifications, and perioperative interventions, can help mitigate symptoms and improve patient-centered outcomes. Future research should prioritize standardized definitions and objective assessment tools, and directly compare surgical approaches in high-quality, multicenter studies.

**Table 3:** Studies related to modifications in treatment practice, perioperative strategies or technical equipment in thyroidectomy aimed at reducing postoperative dysphagia.

Authors	Year	Country	Study Design	Study Period	Indications	Sample	Preoperative Symptoms/Symptom Evaluation	Operative Technique/Treatment Practice	Results
Ben Nun et al. [33]	2006	Israel	R	January 1990– January 2005	Retrosternal goiter	75	Choking, dyspnea	Cervical TT: 68 (91%). Substernal TT: 7 (9%).	Symptomatic improvement.
Almeida et al. [44]	2009	Brazil	C-S	1997–2006	DTC	154	HR-QOL	TT: 100%; ND: 38 (24.7%); RIT: 93 (60.4%).	Better scores: Patients ≤ 45 years, in selective or without ND, RIT < 150 mCi.
Pradeep et al. [46]	2011	India	R	Not specified	Hashimoto’s thyroiditis	271	Tightness in the neck, discomfort in swallowing	Thyroidectomy Group A: 35 patients with HT. Group B: 236 patients with other benign thyroid diseases.	Discomfort in swallowing and tightness in the neck were relieved at 3 months after surgery.
Silva et al. [16]	2012	Brazil	C-S	May 2006– July 2007	DTC (46%), goiter (44%), thyroiditis (3%), other (7%).	308	UADS Questionnaire	Thyroidectomy: 208 OS without IONM, 100 OS with IONM.	Positive impact of IONM: decreasing the prevalence and degree of disturbance of long-term UADS after thyroidectomy. No relation between treatment with iodine therapy, extent of surgery in NMG and the prevalence of UADS. More swallowing complaints in TT than in partial thyroidectomy.
Xu et al. [42]	2012	China	RC	Not specified	Thyroid surgery of unspecified etiology	240	POST severity assessed at 1, 6, and 24 h after extubation	Thyroid Surgery: Group A: 7.0 ETT with saline; Group B: 6.0 ETT with saline; Group C: 7.0 ETT with lidocaine; Group D: 6.0 ETT with lidocaine.	Decrease in severity and incidence of POST in thyroid surgery with the use of smaller-sized ETT combined with IV Lidocaine.
Gal et al. [45]	2013	USA	C-S, R	1992–2008	Well DTC	34	QOL Radiation Therapy Instrument, Head and Neck Companion Module	11 patients only TT 11 patients TT with postoperative RAI 13 patients XRT.	XRT group reported worse chewing, appetite, swallowing, and pain compared to RAI and TT groups. Both RAI and XRT groups experienced significant declines in QOL compared to TT group.
Ryu et al. [41]	2013	Republic of Korea	P, RC	Not specified	Elective thyroidectomy of unspecified etiology	90	Incidence and severity of hoarseness, dysphagia, POST, cough at 2 and 24 h postoperatively	All patients: total intravenous anesthesia with propofol and remifentanil. Group A: 45 patients, cuff pressure to 25 cm H_2_O initially, without adjustment during thyroidectomy. Group B: 45 patients, cuff pressure to 25 cm H_2_O throughout the operation.	Adjusting the endotracheal cuff pressure during thyroidectomy decreased the incidence and degree of POST.
Alkan et al. [36]	2014	Turkey	P	Not specified	Benign multinodular goiter	16	Pre- and postoperatively: Interview for presence of dysphagia, hoarseness, throat obstacle, pharyngeal annoyance and cough during bolus transit, sensation of foreign body in the pharynx. VSLS, CPM EMG, submental EMG single-bolus analysis	Primary TT: Group 1: 8 patients without the use of seprafilm. Group 2: 8 patients with the use of seprafilm between the strap muscles and the laryngotracheal unit.	The use of seprafilm between larynx and strap muscles during TT does not result in any electrophysiological difference regarding swallowing. Anti-adhesive barrier does not have any adverse effects, does not result in foreign body sensation, and can be used safely during thyroid surgery.
Del Rio et al. [38]	2015	Italia	P	Not specified	Benign and malignant of thyroid diseases	80	Self-evaluation of dysphagia to liquids and pain	Traditional thyroidectomy using reusable vs. disposable devices: Group A: BiClamp 150. Group B: Harmonic Focus.	Dysphagia for liquids on a scale from 0 to 10: Group A: 4.5 ± 2.35. Group B: 4.18 ± 2.4. BiClamp is a viable alternative tool with a high security standard and low cost.
Chun et al. [39]	2015	Republic of Korea	P, RC, double-blinded	July 2013–February 2014	Elective thyroid lobectomy of unspecified etiology	64	MDADI, LPS	General anesthesia provided with an LMA or ETI.	The use of LMA in general anesthesia for thyroid surgery has advantages over the ETI in relieving the laryngopharyngeal symptoms, and in decreasing patients’ subjective and objective voice symptoms, reducing the duration of symptoms.
Kim, D. Y. [37]	2015	Republic of Korea	RC, double-blinded	Not specified	Papillary thyroid carcinoma	39	Swallowing Impairment Index	Conventional, open TT: 19 patients without ADM; 20 patients with ADM.	ADM-assisted implants improve post-thyroidectomy scarring and swallowing impairments without prolonging operative time.
Exarchos et al. [43]	2016	Greece	R	September 2012– December 2014	Not specified	118	SIS-6, laryngoscopy	TT: Group 1: Patients who received a single perioperative dose of dexamethasone. Group 2: Patients who did not receive the steroid.	48 h after TT: significantly lower SIS-6 in patients who received perioperative dexamethasone. 1 m after TT: No significant difference in SIS-6 between the dexamethasone and non-steroid groups.
Wang et al. [34]	2016	China	R	December 2012– December 2014	Substernal goiter	27	Not specified	15 patients with laparoscopic thyroidectomy via areola approach; 12 patients with open thyroidectomy via low-neck collar cervical approach.	Laparoscopic thyroidectomy for the treatment of substernal goiter via the areola approach is feasible. There were no cases of hoarseness, dysphagia, lymphatic leakage, dyspnea and death in either group.
Sorensen [3]	2018	Denmark	P, C-C, RC	November 2014– April 2016	Benign nodular goiter	33	Goiter symptom scale of ThyPRO, questionnaire HREM	TT, HT, isthmectomy, lobectomy.	Swallowing symptoms often worsened immediately after surgery but typically showed significant improvement compared to baseline by the 6-month mark. The SCAE increased by 34% after surgery. Esophageal deviation and compression were significantly reduced.
Koo et al. [40]	2019	Republic of Korea	P, RC	June 2016–November 2017	Intraparenchymal thyroid cancer with a size < 2 cm	104	Incidence and severity of hoarseness, dysphagia, POST, cough at 1, 6, 24 and 48 h postoperatively	SERT: Control group: (n = 52) 25 mmHg initial cuff pressure, monitored without adjustment. Adjusted group: (n = 52) with adjustment at approximately 25 mmHg throughout surgery.	No differences in the incidence of dysphagia hoarseness, or cough between the two groups, except for dysphagia and cough at 6 h postoperatively (11.4% in the adjusted group vs. 29.2% in the control group). Therefore, intraoperative monitoring and adjustment of cuff pressure can reduce the incidence of laryngo-pharyngeal complications.
Goswami et al. [47]	2019	USA	R, C	Not specified	Thyroid Cancer Survivors	1743	HRQOL score, online survey regarding clinical history, PROMIS 29 instrument	Surgery and RAI ablation.	High incidence of complications related to surgery and RAI ablation. Postoperative dysphonia, dysphagia, hypocalcemia, and age < 45 years, predicted worse HRQOL scores.

## 5. Conclusions

Post-thyroidectomy dysphagia represents a clinically significant complication with important implications for patient care and surgical planning. Its multifactorial nature underscores the need for individualized perioperative strategies, including careful patient counseling, optimized surgical technique, meticulous perioperative management, and consideration of adjuvant therapies. Emerging minimally invasive and robotic approaches may offer functional and esthetic benefits, but current evidence does not conclusively demonstrate superiority in reducing swallowing dysfunction. Objective and subjective assessments of swallowing, alongside long-term follow-up, are critical for identifying at-risk patients and guiding interventions. By highlighting the clinical relevance of dysphagia and its determinants, this review provides a foundation for future research aimed at optimizing surgical outcomes, minimizing functional morbidity, and improving quality of life for patients undergoing thyroidectomy.

### Difficulties and Limitations

This systematic review was limited by gaps and inconsistencies in the existing literature. Many studies lacked detailed preoperative data, making it difficult to determine whether postoperative changes reflect true improvement or decline. Short follow-up periods and reliance on heterogeneous or author-modified rating scales further restricted assessment of long-term outcomes and comparability across studies. Overall, the absence of standardized evaluation protocols for swallowing function before and after thyroidectomy limits the reliability of pooled findings and underscores the need for uniform assessment methods in future research. According to the ROBINS-I assessment, the overall certainty of evidence was limited by a moderate to serious risk of bias, particularly due to confounding and non-standardized outcome measurement.

Furthermore, although several studies incorporated objective instrumental assessments, their limited number, heterogeneous methodologies, and inconsistent timing of evaluation precluded meaningful quantitative synthesis.

## Figures and Tables

**Table 1:** Summary of risk of bias assessment using ROBINS-I.

ROBINS-I Domain	Risk of Bias	Main Concerns
Bias due to confounding	High	Inconsistent adjustment for age, sex, baseline dysphagia, extent of surgery, nerve injury, anesthesia, and adjuvant therapy
Bias in selection of participants	Moderate	Single-center studies, unclear recruitment strategies, limited use of control groups
Bias in classification of interventions	Low	Surgical procedures generally well defined
Bias due to deviations from intended interventions	Low–Moderate	Limited reporting on perioperative variations and protocol adherence
Bias due to missing data	Moderate	Loss to follow-up and incomplete reporting in longer-term studies
Bias in measurement of outcomes	High	Predominant reliance on subjective, non-standardized questionnaires; limited objective assessment
Bias in selection of reported results	Moderate	Selective reporting of outcomes and time points; omission of absolute prevalence data
Overall risk of bias	Moderate–Serious	Driven mainly by confounding and outcome measurement bias

**Table 2:** Essential features of included in the present systematic review studies related to Dysphagia in thyroidectomy.

Authors	Year	Study Design	Sample	Age (y)	Approaches to Symptom Evaluation	Operative Technique	Follow-Up	Prevalence of Postoperative Dysphagia
Ikeda et al. [8]	2002	P, C	45	41.17	Questionnaire	15 OS, 15 EAT (anterior chest approach), 15 EAT (axillary approach)	3 m	3 m: 5 (33%)
Pereira et al. [9]	2003	R, C-C	60	58	UADS	38 OS (uncomplicated total), 22 OS (near total)	4 y	9 (15%)
Maung et al. [10]	2005	P	41	48	GETS	OS	3 m, 12 m, 1 y	3 and 12 m: globus symptoms did not worsen
Burns & Timon [11]	2007	P	200	48	Questionnaire	OS	3 m, 6 m, >12 m	58 patients with globus pharyngeus preoperatively, and 80% of symptoms resolved postoperatively
Greenblatt et al. [12]	2009	P	116	49	SWAL-QOL questionnaire	OS	12 m	Significant improvements in 8 SWAL-QOL domains. Lower SWAL-QOL scores for 1 patient with unilateral RLNI.
Lombardi et al. [13]	2009	P	110	46.5	AVA, SIS-6, VIS, VSL, MPT	OS	1 w, 1 m, 3 m, >1 y	1 w: 81 (73.6%) 1 m: 70 (63.6%) 3 m: 53 (48.2%) >1 y: 22 (20%)
Lee et al. [14]	2010	P	84	37.6	VHI-10, SIS-6, VSL	41 RS, 43 OS	1 w, 3 m	1 w: VHI-10 Significantly increased in both groups. 3 m: VHI-10 higher in open group. 1 w and 3 m: SIS-6 significantly higher in open group
Lombardi et al. [2]	2012	P	33	44.5	AVA, SIS-6, VIS, VSL, MPT, LEMG	OS	1 m, 3 m	1 m: 2.81 ± 3.63 3 m: 1.65 ± 2.56
Sabaretnam et al. [15]	2012	P, C-C	224	40.5	SWAL-QOL questionnaire	124 OS, 100 without surgery	>6 m	Scores of SWAL-QoL in 12 domains were low and improved significantly after surgery
Silva et al. [16]	2012	C-S	308	45.2	UADS questionnaire	208 OS without IONM, 100 OS with IONM	15–40 m 13–42 m	OS without IONM: 70 (33.6%) Partial: 19 (24.1%), TT: 51 (39.5%) OS with IONM: 22 (22%) Partial: 10 (31.2%), TT: 12 (17.7%)
Tae et al. [17]	2012	P	111	40.78 54.36	Questionnaire, VSL, MVP, VRP	50 RS, 61 OS	1 d, 1 w, 1 m, 3 m, 6 m	1 d: RS: 2.46 ± 2.07 OS: 3.11 ± 2.85 1 w: RS: 1.63 ± 1.86 OS: 1.82 ± 2.18 1 m: RS: 1.94 ± 2.43 OS: 1.91 ± 2.72 3 m: RS: 1.57 ± 1.99 OS: 1.83 ± 2.53 6 m: RS: 0.75 ± 1.30 OS: 1.02 ± 2.02
Lee et al. [18]	2013	C	128	35.7 42.4	QoL symptom scale, AAT, NDII	62 RS, 66 OS	Not specified	RS: better QoL outcomes & reductions in swallowing discomfort.
Jung et al. [19]	2013	RC	86	48.0 51.8	VHI-10, SIS-6, MVP, VRP	42 OS, subplatysmal approach 44 OS, subfascial approach	2 w, 3 m	2 w: Subplatysmal: 2.81 ± 3.02 Subfascial: 1.59 ± 2.37 3 m: Subplatysmal: 1.24 ± 2.16 Subfascial: 0.64 ± 1.12
Hyun et al. [20]	2014	P, C	47	46.05 39.32	SIS-6, Barium videofluoroscopy	24 OS, 23 EAT	3 d, 1 m	3 d: OS: 11.00 EAT: 6.09 1 m: OS: 6.26 EAT: 4.96
Arakawa-Sugueno et al. [1]	2015	P, C	54	25–65	VSL	OS, MIT	7 d, 60 d	7 d: 87% of patients with ALM and 44% with NLM. 60 d: 67% of patients with ALM and 25% with NLM.
Chung et al. [21]	2015	P, C	94	39.8 47.4	MDVP, VRP, GRBAS scale	47 EAT, 47 OS	1 w, 1 m, 3 m, 6 m, 12 m	1 w: EAT: 3.5 OS: 1.2 1 m: EAT: 3.3 OS: 0.4 3 m: EAT: 0.9 OS: 2.9 6 m: EAT: 0.3 OS: 0.6 12 m: EAT: 0.2 OS: 0
Gohrbandt et al. [22]	2016	P	53	52.4	Questionnaire, ultrasonography	OS	1 m, 3 m, 6 m	1 m: 25 (47.2%) 3 m: 12 (22.6%) 6 m: 4 (7.6%)
Kim, W. W et al. [23]	2016	C, RC	229	50.4 38.9	VHI-10, SIS-6, QoL questionnaire	117 OS, 112 RS	32.3 ± 6.3 m	Swallowing impairment: OS: 0.38 ± 0.07 RS: 0.26 ± 0.06
Lee, D. Y et al. [24]	2016	P, C	280	49.5	MDVP, VRP, MVP, GRBAS scale, VHI-10, DHI, VAS	204 conventional OS, 76 transaxillary thyroidectomy	1 w, 1 m, 3 m, 6 m, 12 m	DHI scores: higher in TA than in COS group, (wider flap elevation and injury to the neck muscle affect this result
Elzahaby et al. [25]	2018	P, C	40	32.2 35.4	Self-reported/not specified	20 EAT with UABA, 20 EAT with MACWA	2 m	2 (5%)
Hillenbrand et al. [26]	2018	R	219	58	Questionnaire	OS	>6–18 m (mean 14)	immediately postoperative: 110 (50.2%) <3 m: 16 (7.3%) >3 m: 39 (17.6%) Significant risk in patients with Graves’ disease, carcinoma, in more invasive operation
Liu et al. [27]	2018	C	143	31.70	VHI-10, SIS-6	68 subplatysmal EAT, 75 subfascial EAT	2 w, 3 m, 6 m	2 w: Subplatysmal: 3.11 ± 2.04 Subfascial: 2.21 ± 1.75 3 m: Subplatysmal: 0.97 ± 1.14 Subfascial: 0.73 ± 1.27 6 m: Subplatysmal: 0.76 ± 0.99 Subfascial: 0.59 ± 1.06
Park et al. [28]	2018	P, C	103	48.02	TVQ	49 TT, 54 lobectomy (HT)	1 m, 3 m, 6 m, 12 m	1 m: TT: 11.8 HT: 7.6 3 m: TT: 11.0 HT: 6.2 6 m: TT: 9.3 HT: 5.5 12 m: TT: 8.4 HT: 6.2
Sorensen [3]	2018	P, C-C, RC	33	60	Goiter symptom scale of ThyPRO questionnaire, HREM	TT, HT, isthmectomy, lobectomy	at baseline, 6 m	Swallowing symptoms often worsened immediately after surgery but typically showed significant improvement compared to baseline by the 6-month mark. The SCAE increased by 34% after surgery. Esophageal deviation and compression were also significantly reduced
Tomoda et al. [29]	2018	P	616	49.9	Questionnaire, FBST, SDS	OS	3 d, 1 m, 3 m, 6 m, 1 y	2 d: 75.3% 1 m: 78.9% 12 m: 49.3% 3 d and 12 m: FBST higher in TT compared to lobectomy
Im et al. [6]	2019	P, C	54	47.33 42.64	VFSS, MDHE, MDLE MBSImp score, PTD, LRD	40 TT, 14 volunteers	1 w, 3 m	Swallowing impairment after TT only in pharyngeal swallowing: 35% at 1 w At 3 m 89.3% improvement
Sahli et al. [30]	2019	R	924	51.1	Self-reported/not specified	OS	1–4 w	1 m: 51 (5.5%)
Yu et al. [31]	2019	R	5	46	Self-reported/not specified	OS, MIT	10–20 m	>1 y: 0 (0%)
Cho et al. [5]	2020	P	40	46.8	US evaluation, TVQ score	22 HT, 18 TT	1 m, 3 m, 6 m	12.40 ± 2.28, 9.78 ± 1.93, 7.23 ± 1.90 TT group: higher TVQ score
Jian et al. [32]	2020	P, C	150	38.4 46.56 43.93	CRP	50 total EAT, 50 EAT, 50 conventional OS	6 h, 24 h, 72 h	4.12 ± 1.31 2.02 ± 1.12 3.22 ± 1.69
Costa et al. [4]	2021	C-S	40	49.55 40.75	UADS, TLUS, HBET, MHBDT, MHBDMT	20 OS 20 without surgery	Not specified	Clearing (75%), hoarseness (55%), feeling of bolus in the throat (50%), dry throat (50%)

h: hours, d: days, w: weeks, m: months, y: years.

## Data Availability

No new data were created or analyzed in this study.

## Supplementary Materials

The following supporting information can be downloaded at: https://www.mdpi.com/article/10.3390/medicina62030440/s1, File S1: PRISMA 2020 Checklist [7].

## Abbreviations

AAT	Arm abduction test
ADM	Acellular dermal matrix
ALM	Abnormal laryngeal mobility
AVA	Acoustic voice analysis
C	Comparative
C-C	Case–control
CPM EMG	Cricopharyngeal muscle electromyography
CRP	C-reactive protein
C-S	Cross-sectional
DHI	Dysphagia handicap index
DTC	Differentiated thyroid cancer
EAT	Endoscopically assisted thyroidectomy
ETI	Endotracheal intubation
ETT	Endotracheal tube
FBST	Foreign-body sensation in the throat score
GETS	Glasgow–Edinburgh throat scale
GRBAS scale	Grade of hoarseness (G), roughness (R), breathiness (B), asthenic (A), and strain (S)
HBET	Hyoid bone elevation time
HREM	High-resolution esophageal manometry
HRQoL	Health-related quality of life
HT	Hemithyroidectomy
LMA	Laryngeal mask airway
LPS	Laryngopharyngeal symptom score
LRD	Laryngeal response duration
MACWA	Modified anterior chest wall approach
MBSImp	Modified barium swallowing impairment profile
MDADI	MD Anderson dysphagia inventory
MDHE	Maximal distance of hyoid excursion
MDLE	Maximal distance of laryngeal excursion
MHBDT	Maximum hyoid bone displacement time
MHBDMT	Maximum hyoid bone displacement or maintenance time
MIT	Minimally invasive technique
MIVAT	Minimally invasive video-assisted thyroidectomy
MPT	Maximum phonation time
MVP	Multidimensional voice program
ND	Neck dissection
NDII	Neck dissection impairment index
NLM	Normal laryngeal mobility
OS	Open surgery
P	Prospective
POST	Postoperative sore throat
PROMIS	Patient-reported outcomes measurement information system
PTD	Pharyngeal transit duration
R	Retrospective
RAI	Radioactive iodine
RC	Randomized controlled
RLNI	Recurrent laryngeal nerve injury
RS	Robotic surgery
SCAE	Smallest cross-sectional area of the esophagus
SDS	Self-rating depression scale
SERT	Scarless remote access endoscopic and robotic thyroidectomy
SIS-6	Swallowing impairment score
TLUS	Transcutaneous laryngeal ultrasonography
TT	Total Thyroidectomy
TVQ	Thyroidectomy voice-related questionnaire
UABA	Unilateral axillo-breast approach
VAS	Visual analog scale
VFSS	Videofluoroscopic swallowing study
VHI-10	Voice handicap index-10
VIS	Voice impairment score
VRP	Voice range profile
VSL	Videolaryngostroboscopy
XRT	External beam radiotherapy
